# Use of Carbohydrate (CHO), Gluten-Free, and FODMAP-Free Diets to Prevent Gastrointestinal Symptoms in Endurance Athletes: A Systematic Review

**DOI:** 10.3390/nu16223852

**Published:** 2024-11-11

**Authors:** Karen Montero-Carrasco, Maria Jose Arias-Tellez, Johana Soto-Sánchez

**Affiliations:** 1 Programa de Magíster en Medicina y Ciencias del Deporte, Escuela de Kinesiología, Universidad Mayor, Camino La Pirámide 5750, Huechuraba 8580000, Chile; kmontero.profe@gmail.com; 2 Department of Nutrition, Faculty of Medicine, University of Chile, Independence 1027, Santiago 8380453, Chile; 3 Centro de Biomedicina, Laboratorio de Actividad Física, Ejercicio y Salud, Universidad Mayor, Camino La Pirámide 5750, Huechuraba 8580000, Chile

**Keywords:** sports nutrition, sports performance, gastrointestinal upset, athletes’ preferences

## Abstract

Background: Gastrointestinal symptoms (GISs) can affect the performance of endurance athletes (EAs). This study aims to analyze the efficacy of carbohydrate (CHO), gluten-free, and low-mono-saccharide and polyol (FODMAP) diets in preventing GISs in adult EAs of both sexes. Methods: A systematic search was conducted prior to 30 June 2024 in accordance with the PRISMA statement. We searched for original studies from the last eight years, in English or Spanish, that looked at the effect of CHO, gluten-free, or FODMAP diets on the GISs of EAs. In PubMed, the MeSH (medical subject heading) categories were used. The search was repeated in EBSCO, Google Scholar, and Web of Science. The inclusion criteria were determined using the PICOS framework and the risk of bias in each paper was assessed using the PEDro scale quality criteria checklist (systematic review registration: INPLASY202490080). Results: Of 289 articles identified, only 3.5% met the eligibility criteria. All studies found that GISs are common in EAs. We found that 60% of the articles used an experimental method; moreover, based on 80% of the articles, following a bowel training diet, like CHO, reduced fiber and dairy products, or a low-FODMAP diet, has the potential to reduce gastrointestinal symptoms and improve the athletic performance of EA. Conclusions: We found that low-FODMAP diets, gut training with CHO intake, and decreased fiber and dairy intake may have favorable effects in preventing GISs. No studies support a gluten-free diet in reducing GISs in EAs.

## 1. Introduction

Endurance athletes (EAs) often experience gastrointestinal symptoms (GISs) [1,2] that affect their performance and health [3,4]. Previous findings suggest that up to 70% of EAs, during rest and moderate and vigorous exercise, can have a high frequency and intensity of symptoms [5,6]. These multifactorial symptoms involve mechanical, physiological, and nutritional factors [2,7]. They may occur before, during, or after exercise and manifest as upper symptoms (including nausea, vomiting, and reflux) or lower symptoms (including abdominal pain, bloating, flatulence, diarrhea, and rectal bleeding) [8].

Appropriate nutrition could help with managing the symptomology of GISs. The implementation of popular diets has increased rapidly in recent years due to their perceived ergogenic and health benefits [9,10,11]. A recently published exploratory study found that the self-reported nutritional strategies used more commonly by EAs are those related to different types of carbohydrates (CHOs) (dietary fiber reduction, dairy avoidance, FODMAP diet) [12]. In addition, a recent review [1] showed that the FODMAP diet and employing repetitive gut training using carbohydrates are the most used approaches by Ultra-EAs to alleviate exercise-induced GISs. Interestingly, over recent years, adherence to a gluten-free diet in non-celiac athletes has become increasingly popular [13]. Surprisingly, forty percent of non-celiac athletes report following this diet at least half the time, and 60% have a self-reported “gluten intolerance” [10,14].

CHO is a fuel source oxidized by skeletal muscle tissue during prolonged exercise [15]. The consumption of CHOs during exercise can improve endurance and performance during prolonged exercise (>2 h) [16]. Additionally, previous studies have reported that efficient oxidation is associated with a lower accumulation of CHO in the gastrointestinal tract, reducing the risk of developing GIS discomfort [17,18]. Gastrointestinal training with a high amount of CHO is a strategy that can help athletes improve their tolerance to CHO intake and reduce GISs during exercise [19,20]. This involves the consumption of CHO during training to enhance absorption and utilization during prolonged exercise [16].

Gluten is a complex mixture of proteins present in foods such as wheat, rye, barley, and oats that are incompletely digested by intestinal enzymes [21]. A gluten-free diet is essential for managing symptoms in people diagnosed with celiac disease or gluten sensitivity [22]. This diet has also been popularized among non-celiac athletes [10] due to the belief that it is a healthy and balanced diet [13]. However, there is no scientific evidence that this diet supports improved mental or physical performance in healthy people [23].

FODMAPs are CHOs that are poorly absorbed and highly fermentable by the intestinal flora, and are present in fruits, vegetables, cereals, milk, dairy products, legumes, and sweeteners [24]. The main types of FODMAPs are fructose, lactose, oligosaccharides, and polyols, each with a distinct action mechanism [25]. The low-FODMAP diet [24,26,27] involves the reduced consumption of fermentable short-chain CHOs and is used in people with non-specific digestive symptoms such as irritable bowel syndrome [28,29]. In athletes who perform strenuous exercise, undigested molecules can increase the osmotic load in the small intestine, leading to increased stool volume or diarrhea [30]. The low-FODMAP diet is associated with improved GISs in 50–80% of patients. However, following this diet is not often easy due to an unintuitive and very restrictive list of foods that can increase the risk of deficiencies and imbalances in the microbiota [31].

The relationship between nutritional interventions and the maintenance or alteration of intestinal integrity is still unclear. Even though the intake of CHOs could benefit the performance of athletes, their impact on GISs is yet unknown, making it difficult to develop recommendations [32]. In addition, gluten-free and low-FODMAP diets are popularly suggested to improve gastrointestinal health [33]. Based on previous reviews, it is evident that a substantial number of athletes are not diagnosed with a clinical condition necessitating a gluten-free diet to prevent gastrointestinal issues [34]. Additionally, athletes who adhere to a gluten-free diet inadvertently reduce their intake of high-FODMAP foods, effectively reducing gastrointestinal symptoms [11,35]. This approach depends on the athletes’ characteristics and the severity of gastrointestinal issues [36]. Given this context, the following question arises: Are CHO, gluten-free, and low-FODMAP diets effective in mitigating GISs in EAs? Consequently, this review aims to analyze the efficacy of CHO, gluten-free, and low-FODMAP diets in preventing GISs in adult EAs of both sexes.

## 2. Materials and Methods

### 2.1. Search Strategy

A systematic search was carried out for articles published before June 30, 2024, following the criteria of the PRISMA declaration [37]. We used generic terms to identify all studies addressing the efficacy of CHO, gluten-free, and low-FODMAP diets in preventing GISs in adult EAs of both sexes. The search criteria were (((“Diet, Carbohydrate-Restricted”))) OR (“Diet, Gluten-Free” AND (“Gastrointestinal Diseases”)) AND (“Athletes”). In PubMed, the MeSH (Medical Subject Heading) terms were used. The same search strategy and combination of terms was repeated in EBSCO, Google Scholar, and Web of Science. A PRISMA flow diagram was used to show the search strategy steps for this systematic review.

### 2.2. Inclusion and Exclusion Criteria

The inclusion criteria were determined using the PICOS (population, intervention, comparators, outcomes, study design) model (Table 1).

A researcher (KNMC) reviewed, in detail, whether the articles met the inclusion criteria established in two phases: (a) reading the title and abstract and (b) reading the full text of the articles included in the previous phase.

The exclusion criteria were (a) narrative or systematic reviews; (b) studies on athletes diagnosed with celiac disease; (c) articles related to non-athletes or other sports disciplines; and (d) studies that included results of the use or intake of CHO, gluten, and FODMAP diets not related to GISs.

### 2.3. Quality Assessment of Studies

The risk of bias and quality in each paper was assessed by KNMC using the (Table 2) PEDro Scale (Physiotherapy Evidence Database) checklist [38] containing 11 criteria (eligibility, random allocation, concealed allocation, baseline comparability, blind subjects, blind therapies, blind assessors, adequate follow-up, intention-to-treat analyses, between-group comparations, and point estimates and variability). A score > 6 was considered acceptable to be considered in this review. Further details regarding the PEDro Scale methodology can be found elsewhere [38].

### 2.4. Process of Extraction of Information

After the inclusion criteria and PEDro scale checklist were applied, information on the author and year of publication, objective, type of diet, methodology, results, and conclusion were extracted by two authors (KNMC, MJAT), and the systematic review was registered at https://inplasy.com/inplasy-2024-9-0080/ (accessed on 18 September 2024), identifier INPLASY202490080.

## 3. Results

**Figure 1:**
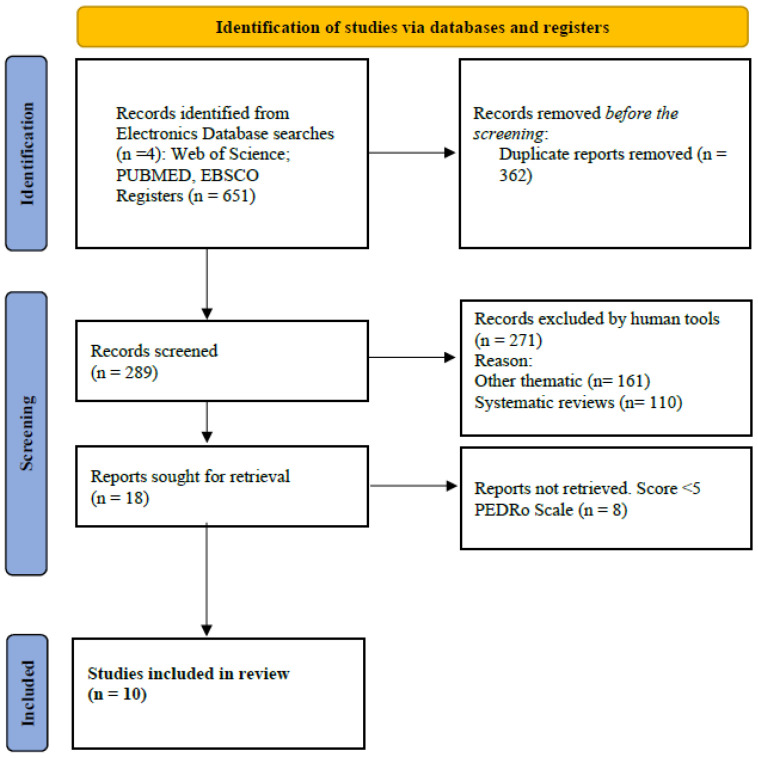
Flow diagram according to PRISMA 2020.

A total of 651 articles were identified, of which 55% (*n* = 362) were duplicated in the different databases. In this phase, 289 articles were selected by reading the title and abstract. Moreover, 93% (*n* = 271) were excluded because they were systematic reviews or did not include results that would allow for an analysis of the efficacy of CHO, gluten-free, and low-FODMAP diets in preventing GISs in adult EAs of both sexes. The remaining 18 articles were evaluated using the PEDro Scale [38]. Of these, eight articles were excluded because they did not score ≥6 on the PEDro Scale. The PRISMA flow diagram (Figure 1) shows the steps of the search strategy and the 10 articles considered for this systematic review.

Table 3 summarizes the selected articles. Of the studies included in this review, 60% included males and females, and 90% involved trained or recreational adult runners. One study incorporated EAs from a discipline other than running [47].

Of the 10 articles, 60% used an experimental method to search for results that supported the benefit of dietary interventions based on a CHO diet, gluten-free diet, or low-FODMAP diet. In contrast, the other 40% implemented validated questionnaires or diary records to collect background information on the strategies used to mitigate gastrointestinal discomfort in EAs. It is worth mentioning that two of the studies used dietary records as a tool that was complementary to the experimental intervention. In addition, for determining the incidence and severity of GISs related to exercise, scales or questionnaires were used. The instruments used by the authors were (i) a 10-point Likert-type rating scale (*n* = 3), (ii) the Syndrome Severity Scoring System (*n* = 1), (iii) web-based questionnaires (*n* = 1), (iv) the visual analog scale (*n* = 3) [48], (v) a questionnaire to assess GI symptoms exercise-induced (without Likert-scale items) (*n* = 1), and (vi) a gut comfort questionnaire (validate by prior authors) (*n* = 1).

Finally, 80% of the articles concluded that an endurance athlete’s daily intake of a bowel training diet involving CHO (*n* = 3), a decrease in fiber and dairy products (*n* = 1), or low-FODMAP foods (*n* = 4) could reduce GISs and improve sports performance. Studies on using a gluten-free diet to mitigate GISs, which also met our selection criteria, were not found.

## 4. Discussion

The results of the present systematic review show that the most successful strategies to reduce GISs are gastrointestinal training using CHO and the introduction of a low-FODMAP diet. However, no scientific evidence supports using gluten-free dietary strategies to mitigate such symptoms in EAs. We found a result [12] that suggested mitigating GISs by avoiding dairy and fiber. This finding is not a widely used solution, given that no other reviews or studies were found for or against it. The elimination of dairy and fiber and the reduction of gastrointestinal symptoms may not solely be attributed to lactose-containing foods and increased intestinal transit. These effects can also be concealed by symptoms caused by other FODMAPs. Due to the development of recommendations that simultaneously improve athletic performance by attenuating gastrointestinal symptoms, which is necessary for trainers and sports nutritionists, our results emphasize the need to increase the number of controlled clinical trials that compare the efficacy of CHO and FODMAP diets on managing the symptomology of GISs in endurance disciplines.

Previous research has suggested that consuming carbohydrates during exercise can lead to gastrointestinal distress and diarrhea in athletes [49]. As a result, CHO training during exercise could serve as a preventive nutritional strategy. However, in this review, we only found two studies that concluded that CHO training reduces intestinal malabsorption and GISs in exercise-associated gastrointestinal distress among EAs. A higher intake of CHO during exercise could increase the severity of GISs and lead to intolerance, particularly during high-intensity exercise [45]. A linear relationship between CHO intake and GISs based on the distance (kilometers) run has yet to be observed [42]. Consequently, sports nutritionists must take this evidence into consideration when they plan to address GISs in EAs using CHO training, and more studies are also necessary to clarify the effect of CHO training in different endurance disciplines and develop recommendations grams per weight before and during exercise for GIS management.

Our findings align with the results previously shown by Devrim-Lanpir et al. [11], who investigated the impact of five different dietary approaches (i.e., vegetarian, high-fat, intermittent fasting, gluten-free, and low-FODMAP) on the performance and health aspects of EAs. They concluded that a low-FODMAP diet may be more beneficial than a gluten-free diet in athletes without gluten intolerance. Nevertheless, due to the recommendation for a low-FOD dairy intake (g/d) not being determined in EAs, the results do not propose this diet as a definitive strategy. Interestingly, a recent study [14] showed that up to 80% of athletes frequently remove sources of lactose compared to other high-FODMAP foods to improve their GISs. This finding indicates that a low-FODMAP diet may not be sustainable over the entire life course and that restricting foods could inadvertently cause more problems (for example, low intake of essential nutrients such as calcium and imbalance in the microbiota) [50]. However, it could be considered a strategy for the following competencies and highlights the need to evaluate groups of foods on exercise-induced GI symptoms in athletes to confirm low-FODMAP diets as a solution to EAs’ intestinal disorders. Low-FODMAP could also be considered a strategy to reduce GISs in EAs.

The increasing popularity of gluten-free diets in EAs without celiac disease has been discussed extensively [10,13]. Surprisingly, almost 40% (more females than males) of EAs have declared gluten elimination from their daily food intake with the objective of GIS reduction [13,34]. In agreement with our findings, Lis and Cols [51] assert that such a diet would not have a beneficial effect on performance, gastrointestinal health, or well-being; the reduced integrity of the GI barrier in non-celiac athletes is a consequence of exercise intensity and splanchnic hypoperfusion, and not of damage to the intestinal barrier (celiac disease). Interestingly, reducing FODMAPs, rather than gluten, may improve symptoms [36,51], which could be explained by the decrease in the fructans and galactic-oligosaccharides (FODMAPs) present in wheat [52]. Adopting a gluten-free diet when not medically necessary for non-celiac athletes may lead to unintended consequences such as reduced energy levels and a lack of important nutrients like B vitamins, fiber, and iron, which are crucial for optimal sports nutrition. Additionally, it can also result in a higher financial burden and psychosocial implications [34,51]. Adequate and personalized advice is necessary before adopting a gluten-free diet. When planning a gluten-free diet for EAs, it is important for sports nutritionists to consider this evidence.

A strength of this study is the application of the PEDro Scale [38] when selecting high-quality articles for this review. However, a limitation of this study may be the restriction of the search period to only the last 8 years, which could explain the absence of studies on using a gluten-free diet to treat GISs.

## 5. Conclusions

In summary, a personalized gastrointestinal training plan can benefit EAs, helping them achieve their nutritional and athletic goals more quickly. Dietary strategies such as CHO training during exercise and reducing dietary FODMAP intake could help mitigate GISs in adult EAs of both sexes. However, these strategies need more scientific evidence to support their effectiveness. In conclusion, current nutritional recommendations for athletes do not include adequate plans to reduce GISs [10,53]. Thus, randomized clinical trials are required to determine the efficacy of CHO and FODMAP diets and to determine the guidelines for sports nutrition and GIS management.

## Figures and Tables

**Table 1:** PICOS model for determination of inclusion criteria.

	Topic	Criteria
P	Population	Adult endurance athletes or runners with GISs of both sexes.
I	Intervention	Use of CHO, gluten-free, and low-FODMAP diets in preventing GISs or the application of a dietary questionnaire for determining the incidence and severity of GISs related to exercise.
C	Comparators	None/placebo.
O	Outcomes	GI reports (self-reported gut symptoms).
S	Study design	Original studies from the last eight years (cross-sectional studies, randomized controlled trials, crossover trials, case studies), written in English or Spanish, available to the authors as a full text that includes results about the relationship between the use or intake of CHO, gluten-free, and low-FODMAP diets with GISs in EAs or runners.

**Table 2:** Scientific quality of studies according to PEDro Scale [38].

PEDro Scale	Costa et al. [39]	Lis et al. [40]	Miall et al. [41]	Wiffin et al. [25]	Hoogervorst et al. [42]	Parnell et al. [43]	Gaskell et al. [44]	Rauch et al. [45]	Etxebarria et al. [46]	Scrivin et al. [12]
Eligibility criteria	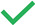	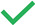	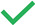	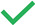	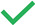	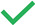	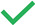	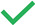	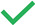	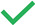
2. Random allocation	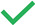	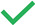	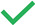	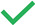	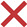	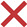	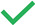	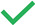	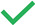	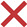
3. Concealed allocation	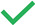	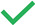	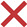	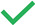	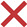	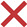	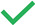	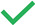	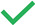	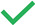
4. Baseline comparability	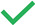	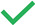	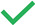	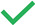	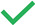	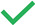	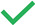	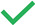	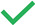	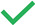
5. Blind subjects	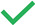	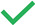	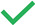	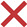	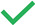	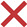	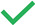	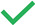	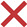	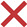
6. Blind Therapists	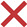	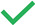	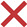	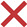	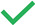	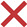	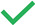	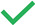	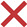	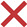
7. Blind assessors	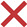	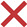	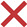	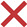	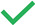	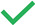	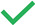	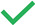	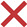	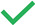
8. Adequate follow-up	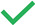	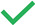	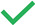	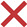	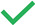	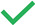	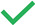	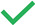	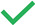	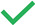
9. Intention-to-treat analysis	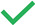	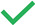	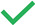	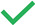	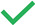	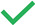	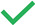	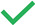	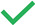	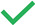
10. Between-group comparisons	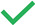	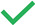	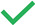	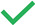	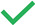	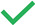	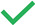	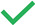	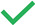	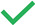
11. Point estimates and variability	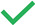	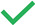	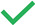	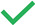	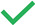	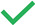	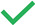	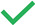	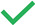	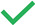
TOTAL	8/10	9/10	7/10	6/10	8/10	6/10	10/10	10/10	7/10	7/10

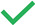
 contains the criteria; 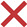 does not contain the criteria.

**Table 3:** Studies examining the efficacy of gluten-free, FODMAP, and CHO-free diets in the prevention of GISs in adult endurance athletes of one or both sexes.

Author	Objective	Methodology			Population	Results	Conclusions
		Characteristics of Diet	Questionnaire or Scale to Quantify GIS	Intervention			
Costa et al. [39]	To determine the impact of two-week bowel training with CHOs on gastrointestinal status and sports performance.	CHO diet.	A 10-point Likert-type rating scale.	The bowel challenge test was applied to the initial condition (GC-1) and repeated after 2 weeks of bowel training (GC2). Bowel training intervention with (a) CHO-S gel disc. (b) food with CHO-F. (c) Placebo (PLA).	25 (10 women) recreationally competitive endurance and ultra-endurance runners.	GIS reduction in GC2: CHO-S (60%; *p* = 0.008) CHO-F (63%; *p* = 0.046) PLA (*p* ≤ 0.05). Improvement in the distance test in GC2: CHO-S (5.2%). CHO-F (4.3%). But not in PLA (−2.1%). (test × time: *p* = 0.009).	Two weeks of gut training with CHO-S and CHO-F improved GISs compared to PLA.
Lis et al. [40]	To investigate the effects of an LFOD in runners with a history of non-clinical exercise-associated GIS.	FODMAP diet.	A 10-point Likert-type rating scale.	LFOD diet + 2 session of 5 × 1000 m running sessions + 7 km threshold race. One day of stomach lavage HFOD + 2 5 × 1000 m running sessions + 7 km threshold run. GI symptoms was measured during exercise and daily.	11 recreationally (6 women) competitive runners.	During LFOD, a significantly lower AUC was observed for daily GISs. The daily GISs that decreased significantly were flatulence (*p* < 0.001), urge to defecate (*p* = 0.04), loose stools (*p* = 0.03), and diarrhea (*p* = 0.004). No significant differences in exercise symptoms or DALDA responses were observed between diets (*p* > 0.05).	LFOD may be a beneficial intervention to minimize daily GISs in runners with exercise-related discomfort.
Miall et al. [41]	To determine whether two weeks of repetitive bowel challenge during running can reduce exercise-associated GISs and CHO malabsorption.	CHO diet.	10-point Likert-type rating scale.	Initial bowel challenge test (GC1) with 30 g CHO (2:1 glucose/fructose, 10% *w*/*v*) every 20 min and subsequent randomization for a two-week repetitive bowel challenge (GC2) intervention with a) 90 g CHO, b) placebo (PLA).	18 (8 women) recreationally competitive endurance and ultra-endurance runners.	Reduction of intestinal discomfort, total upper and lower symptoms, and nausea in GC2 with CHO but not in PLA. The effort series distance was greater in GC2 compared to GC1 alone in CHO.	Repetitive bowel challenge with a gel disc formulation of CHO improves GISs and reduces CHO malabsorption.
Wiffin et al. [25]	To assess whether a short-term LFOD improves exercise-related GISs and perceived exercise capacity in recreational runners.	FODMAP diet.	Syndrome Severity Scoring System.	Participants were randomly assigned to an LFOD or HFOD. A questionnaire was adapted from the Questionnaire to assess Irritable Bowel.	16 (10 women) healthy volunteer recreational runners.	The overall IBS-SSS score was significantly reduced in the LFOD condition. Perceived exercise frequency and intensity improved significantly after a short-term LFOD approach compared to HFOD. No significant differences were reported between dietary conditions for plasma I-FABP (*p* > 0.05).	Recreational athletes who implement a short-term LFOD diet may experience benefits in exercise-related GISs.
Hoogervort et al. [42]	To investigate differences in exercise-related GISs between groups of runners of different distances.	Self-reported diet by questionnaire.	Web-based questionnaires (during exercise and post 12 h).	Data from the Exercise Food and Liquid Questionnaire (FFEQ) were collected before exercise (one hour before starting), during exercise, and 12 h after competition.	527 runners of eight races in the Netherlands, i.e., six marathons (42.195 km) and two ultra marathons (60 km and 120 km).	For all runners, belching was inversely correlated with fiber intake (r = −0.19, *p* = 0.022) and diarrhea with CHO intake (r = −017, *p* = 0.040). Also, at the 120 km distance, the opposite relationship was found between defecation frequency and CHO intake (r = −0.730, *p* = 0.040). The most common complaints during the race were the urge to urinate, muscle cramps, and belching. After the race, the most common complaints were muscle cramps, flatulence, and bloating.	Food choices can influence exercise-related GISs. The fiber and CHO intake were weakly and inversely related to belching and diarrhea. In addition, CHO intake could reduce the defecation frequency at the 120 km distance.
Parnell et al. [43]	To assess voluntary pre-exercise food restrictions related to GISs and differences related to gender, age, performance level, and event.	Voluntary dietary restrictions.	Exercise-induced GI symptoms questionnaire (without scale).	A questionnaire was applied to determine pre-race dietary restrictions and GISs.	388 runners (210 women).	Runners regularly avoided meat, dairy, fish/seafood, poultry, and high-fiber foods. Common GISs included stomach pain/cramps, intestinal pain/discomfort, side pain/stitch, urge to have a bowel movement, and bloating. The prevalence of GISs was higher in younger athletes. The incidence of diarrhea increased with running distance.	Identifying food avoidance trends will guide future clinical trials designed to identify specific foods that endurance runners can consume to minimize GISs and optimize performance.
Gaskell et al. [44]	To determine the effects of HFOD and LFOD diets before stress heat stress on gastrointestinal integrity, function, and symptoms.	FODMAP diet.	Visual analog scale (mVAS) GIS assessment tool.	Double-blind crossover study. On HFOD and LFOD consumption before completing 2 h of running at 60% VO2 max at an ambient temperature of 35 °C. GISs were determined before exercise, every 15 min during and after recovery.	18 endurance runners (8 women).	A higher AUC concentration of H2 in the breath was observed during HFOD compared to LFOD. HFOD showed a higher severity of GISs than LFOD (before exercise). No differences in plasma cortisol concentration were observed between the diets.	HFOD diet coupled with an HFOD recovery drink after exertional heat stress may exacerbate CHO malabsorption and GIS severity compared to an LFOD diet.
Rauch et al. [45]	To feed tolerance while consuming different carbohydrate regimes during steady-state running.	CHO diet.	Visual analog scale (mVAS) GIS assessment tool.	An incremental exercise test to exhaustion and one of three 3 h steady-state running protocols involving a carbohydrate feeding regime (76–90 g/h). The GIS scale was measured during the endurance exercise test.	28 competitively trained male endurance and ultra-endurance runners.	In response to CHO feeding interventions (90 g/h 2:1 glucose/fructose formulation), 38% of participants showed responses of malabsorption. Greater severity of GISs was observed with higher intakes of CHO (90 vs. 76 g/h) during steady-state exercise and high-intensity exercise.	A higher intake of CHO during exercise can increase the severity of GISs and lead to intolerance.
Etxebarria et al. [46]	To explore whether an athlete’s typical nutritional intake influences any gastrointestinal disturbance induced by high-intensity exercise in heat.	Daily typical nutritional intake.	A gut comfort questionnaire (15 questions, prior to testing and 72 h post each trial) and GIS discomfort questionnaire (a subset of eight questions, 1 h postexercise).	Athletes completed two tests in random order of 15 min at different intensities on a treadmill under conditions of 35 °C (heat) and 21 °C (thermoneutral) and self-reported gut symptoms 1 h and 72 h postexercise. Daily nutritional intake was recorded by the participants over a period of 8 consecutive days.	12 well-trained male endurance athletes.	Starch and fiber intakes were positively associated with 1 h postexercise symptoms (r = 0.57–0.66 [0.09, 0.89]). A moderate and inverse association between GIS 72 h postexercise and CHO intake in the 24 h leading up to the thermoneutral trial (r = −0.55 to −0.61 [0.05, −0.88]). The heat condition induced large increases in biomarker concentrations compared to baseline, but induced mild GISs.	The type of CHO intake on the typical nutritional intake can partly explain the induced or attenuation of GISs induced by moderate- and high-intensity exercise under both heat and thermoneutral conditions.
Scrivin et al. [12]	To investigate the GIS associated with self-reported exercise by endurance athletes and the associated strategies to control symptomatology.	Self-reported diet by questionnaire.	Visual analog scale (mVAS) GIS assessment tool.	Athletes completed a validated online (web) questionnaire and identified when GISs were most frequent around training or competitions. Participants reported the severity of each symptom before, during, and after exercise.	137 adult endurance athletes (different disciplines) with a history of GIS.	The incidence of GISs was higher during training and competitions. The most popular strategies to reduce GISs were reducing dietary fiber, eliminating dairy, and LFOD. The most successful dietary strategies were reduced dietary fiber, low-FODMAP diets, dairy-free diets, and increased CHO.	The most commonly reported successful dietary strategies for managing GISs were a reduction in dietary fiber, a low-FODMAP diet, a dairy-free diet, and increased CHO.

CHO: carbohydrates, CHO-F: food, CHO-S: gel disc, FODMAPs: fermentable diet oligo-, di- and mono-saccharides and polyols, GIS: gastrointestinal symptoms, HFOD: high-FODMAP diet, LFOD: low-FODMAP diet.

## Data Availability

The original contributions presented in the study are included in the article, further inquiries can be directed to the corresponding authors.
